# Non‐epileptic paroxysmal events in Rett syndrome: A systematic review of case‐based and observational evidence

**DOI:** 10.1111/dmcn.70093

**Published:** 2025-11-24

**Authors:** Natasha Bhatti, Daniel E. Lumsden

**Affiliations:** ^1^ East Kent Hospitals University NHS Foundation Trust, Kent and Canterbury Hospital Canterbury Kent UK; ^2^ Complex Motor Disorder Service, Evelina London Children's Hospital London UK; ^3^ Research Department of Early Life Imaging School of Biomedical Imaging and Engineering Sciences, King's College London London UK

## Abstract

**Aim:**

To systematically review and characterize the spectrum of non‐epileptic paroxysmal events in individuals with Rett syndrome (RTT).

**Method:**

We conducted a descriptive systematic review of observational evidence. Searches were conducted across the PubMed, Embase, and OVID databases for studies published from January 1962 to September 2024. Eligible studies included case reports, case series, cohort studies, and small clinical trials that described non‐epileptic events in individuals with clinically or genetically confirmed RTT. Data were extracted on study design, participant characteristics, and event types. Events were categorized into respiratory, neuromotor, and behavioural domains.

**Results:**

Sixty‐two studies met the inclusion criteria, representing a wide age range of individuals with RTT. The most frequently reported paroxysmal non‐epileptic events were respiratory disturbances, including breath‐holding and hyperventilation, followed by vacant spells, involuntary movements, and behavioural episodes such as agitation and inappropriate laughter. Discrepancies were noted between the diagnostic attribution of clinician‐reported and family‐reported events. The term ‘Rett episodes’ was used by a minority of authors.

**Interpretation:**

A wide range of non‐epileptic paroxysmal events requiring different treatment approaches are experienced by patients with RTT. Enhancing clinician awareness and developing clearer diagnostic frameworks are key to improving classification accuracy and preventing unnecessary treatment.

## Graphical abstract

This systematic review identifies and categorizes the spectrum of non‐epileptic paroxysmal events in Rett syndrome. Respiratory disturbances, behavioural episodes, and motor events were the most commonly reported. Improving clinician awareness and diagnostic clarity is key to avoiding unnecessary treatment and enhancing quality of life for individuals with Rett syndrome.

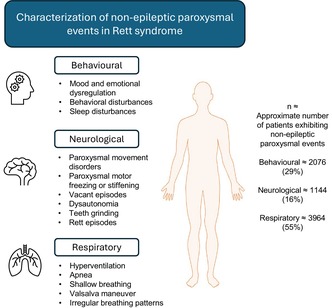

**Abbreviation**
RTT: Rett syndrome

**What this paper adds**

- Non‐epileptic paroxysmal events in Rett syndrome are common, spanning respiratory, motor, autonomic, and behavioural domains.
- Misclassification as epileptic seizures is frequent, with video electroencephalography studies showing that many events are non‐epileptic.
- Case reports, despite heterogeneity, provide consistent evidence of the clinical spectrum of these events.
- Greater diagnostic clarity is essential to avoid unnecessary antiseizure treatments.

Rett syndrome (RTT) is a severe neurodevelopmental disorder primarily affecting females, which is commonly associated with variations in the *MECP2* gene.^1^ Diagnostic criteria for RTT require a period of regression followed by stabilization, with core features including: (1) partial or complete loss of hand skills, (2) partial or complete loss of speech, (3) gait disturbances, and (4) stereotypic hand movements.^2^ Supportive features often seen in atypical cases include breathing irregularities, bruxism, and intense eye contact for communication. Individuals not fulfilling all four core criteria but demonstrating sufficient minor criteria may be considered to have atypical RTT.

Epilepsy affects approximately 70% to 90% of individual with RTT.^3^, ^4^, ^5^ The seizure types experienced are variable. Onset is typically infancy to late childhood and seizures frequently become less severe with age; however, in a minority, they remain resistant to treatment.^6^, ^7^, ^8^

Individuals with RTT also frequently experience non‐epileptic paroxysmal events, as supported by video telemetry studies.^8^ The term ‘Rett episodes’ has been used variably in reference to non‐epileptic events.^9^, ^10^ Despite common use by the community with RTT, a precise clinical characterization of Rett episodes remains unclear, and the differentiation from other non‐epileptic paroxysmal phenomena is imprecisely delineated. Paroxysmal episodes are a common reason families seek specialist input.^11^ Clear characterization of non‐epileptic events is essential to prevent misdiagnosis and inappropriate treatment.

This systematic review aimed to explore the spectrum of non‐epileptic paroxysmal events in individuals with RTT and clarify the phenomenological distinctions between these events and epileptic seizures. We aimed to identify and characterize the range of non‐epileptic paroxysmal events reported in RTT, including behavioural, autonomic, and motor phenomena commonly mistaken for seizures, while establishing which events are most frequently observed to provide a clearer understanding of their prevalence and patterns.

## METHOD

This was a descriptive systematic review of observational evidence, including case reports, case series, cohort studies, and small clinical trials. Preferred Reporting Items for Systematic reviews and Meta‐Analyses (PRISMA) guidelines were followed as a reporting framework. A literature search strategy was implemented across PubMed, OVID, and Embase using a combination of keywords and terms such as ‘Rett syndrome’, ‘non‐epileptic paroxysmal events’, and ‘seizure’ (full details in Table S1). The search was limited to studies published from January 1966 to September 2024. Inclusion criteria consisted of observational studies, case reports, cohort studies, case series, and clinical trials published from January 1966 to September 2024, explicitly reporting non‐epileptic paroxysmal events in patients clinically or genetically diagnosed with RTT. Exclusion criteria were studies focusing exclusively on epileptic events, research involving animal models, studies limited to *MECP2* duplication syndrome only, reviews, editorials, opinion pieces, and conference abstracts. Data were not extracted on stereotypies because these have been well characterized and reported in individuals with RTT. All retrieved references were imported into a citation management software and duplicates were removed. Both authors independently conducted the screening process, first evaluating titles and abstracts, followed by a full‐text review for eligibility. Discrepancies between reviewers were resolved through discussion. Data extraction focused on study characteristics, participant demographics, and types of non‐epileptic paroxysmal events (Table S2).

The search was rerun in May 2025, immediately before manuscript submission, to capture the most up‐to‐date studies and avoid drawing conclusions from outdated evidence.

Language restrictions were not applied at the search stage to reduce the risk of language bias. Where non‐English studies were identified, translated abstracts or full‐text translations were used as needed.

The primary research question guiding this review was: What non‐epileptic paroxysmal events have been described in individuals with Rett syndrome? Specifically, we sought to: (1) characterize the spectrum of reported non‐epileptic events, (2) describe their frequency and phenomenology, and (3) identify where misclassification between epileptic and non‐epileptic phenomena occurred.

Because of the heterogeneity of study designs and outcome definitions, a meta‐analysis was not conducted. Events were categorized according to clinical presentation (e.g. neuromotor phenomena, respiratory dysfunction, and behavioural disturbances) and results were tabulated accordingly. Where possible, patient numbers were pooled across studies to provide a descriptive aggregated estimate of event prevalence. Given the heterogeneity of study designs, case definitions, and outcome measures, pooling was undertaken solely for descriptive purposes rather than for inferential comparison or meta‐analysis. Discrepancies between clinician‐reported and family‐reported data were explicitly highlighted.

### Statistical analysis

Descriptive statistical analysis was performed using SPSS v29 (IBM Corp., Armonk, NY, USA). For the synthesis of case reports and case series, we applied the methodological framework described by Murad et al.^12^ (details provided in the Table S3). We also adhered to principles of good scholarship in systematic reviews as outlined by Kolaski et al.,^13^ including transparent eligibility criteria, consistent data extraction, and critical appraisal of study quality.

This systematic review was conducted in accordance with a prespecified protocol registered on the PROSPERO database (ID: 1077319).

## RESULTS

**Figure 1:**
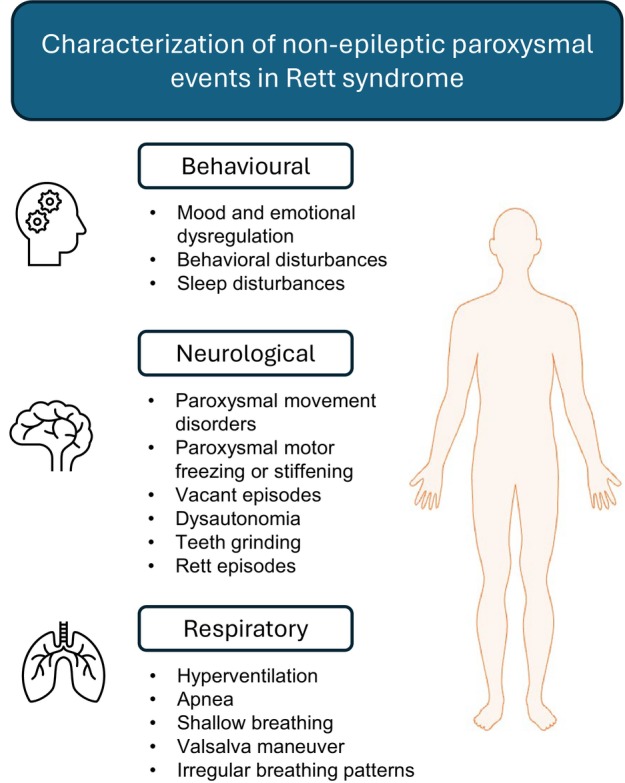
Classification of non‐epileptic paroxysmal events in Rett syndrome. The diverse range of non‐epileptic paroxysmal events observed in Rett syndrome can be categorized into behavioural, neurological, and respiratory manifestations. Behavioural events include mood dysregulation and sleep disturbances, neurological symptoms encompass movement disorders, dystonia, and vacant episodes, while respiratory disturbances notably involve hyperventilation, apnoea, and irregular breathing patterns.

Literature searches identified a total of 4715 studies, with 190 selected for full‐text review (Figure S1). Sixty‐two studies were subsequently included in this systematic review. The sample sizes of these studies ranged from single‐case reports to large cohort studies. A summary of findings is presented in Table 1 and Figure 1.

**Table 1:** Summary of non‐epileptic paroxysmal episodes in individuals with Rett syndrome.

Body system		Event description	Clinician‐reported (participants/studies)	Family‐reported or carer‐reported (participants/studies)
Neurological episodes	Altered consciousness and awareness	Vacant spells and altered consciousness with staring	107/4	531/4
		Paroxysmal activity (EEG abnormalities not consistent with epileptic seizures)	14/4	–
		Non‐epileptic seizures (details of phenomenology not provided)	334/2	–
	Generalized body movements	Paroxysmal dystonia and dystonic posturing	51/2	–
		Tremor	4/2	–
		Myoclonus	5/2	–
		Period limb movements and jerking movements	21/5	–
		Stiffening (not related to dystonia)	3/2	NS
		Drop episodes	NS/1	–
		Other motor abnormalities (unspecified)	NS/1	–
	Craniocervical movements	Involuntary eye movements	NS/2	–
		Head turning	NS/1	–
		Grimacing	½	–
		Teeth grinding	20/3	53/2
Respiratory and autonomic episodes		Respiratory dysrhythmia (irregular breathing)	45/5	NS/1
		Hypopnoea	15/5	–
		Valsalva breathing	41/3	–
		Air swallowing	20/2	82/2
		Apnoea and cyanosis	1092/26	507/9
		Hyperventilation	740/28	217/6
		Other breathing abnormalities (e.g. mouth breathing)	1096/4	93/2
		Dysautonomia	16/4	
Behavioural episodes		Episodic behavioural changes, including screaming, crying, laughter spells (day or night)	96/8	1012/5
		Low mood	2/1	465/2
		Sleep disturbances	11/3	371/5
		Rett episodes/attacks	–	66/1
		Self‐injurious behaviour	–	53/2

Abbreviations: EEG, electroencephalography; NS, not specified.

### Paroxysmal respiratory dysfunction in Rett syndrome

Breathing abnormalities were commonly reported in RTT. Apnoea and breath‐holding episodes associated with cyanosis were documented extensively, reported by clinicians in 1092 patients across 26 studies and by families in 507 patients across nine studies (Tables 1). Episodic hyperventilation was reported by clinicians in 740 patients (28 studies) and families in 217 patients (six studies). Episodic Valsalva breathing was specifically noted in 41 patients in three clinician‐reported studies.^14^, ^15^, ^16^ Air swallowing related to respiratory disturbances was noted across four studies (two clinician‐reported and two family‐reported).

Breathing irregularities persisted into adulthood, for example, they were present in 30 of 50 adults, including apnoea in 27 cases (54%) and hyperventilation in 12 (24%).^17^ Cross‐sectional observations from 56 individuals with RTT aged 2 to 35 years suggested a trend to forced and apneustic breathing in early childhood, with Valsalva breathing becoming more prominent into adulthood.^14^ Similar findings were noted in an earlier observational study of 14 individuals with RTT.^18^ Changes in motor activity (decreases or increases) were commonly described as occurring during episodes of disrupted breathing.^14^, ^18^

### Neuromotor and movement domains

Paroxysmal dystonia and dystonic posturing was reported in 51 patients across two studies (Table 1). Life‐threatening episodes of paroxysmal dystonia resulting in airway compromise were reported in three children and young people with RTT.^19^ Episodic myoclonus was less frequently reported,^20^, ^21^ as was tremor (usually in the context of non‐epileptic episodes) (Table 1).^22^ A range of paroxysmal craniocervical and eye movements was also identified, most commonly bruxism.

### Autonomic and behavioural disturbances

The paroxysmal psychiatric and behavioural disturbances reported included episodic agitation, panic attacks, abrupt behavioural changes, and emotional dysregulation (Table 1). Episodic agitation and panic attacks, including screaming, crying, and laughing spells (day or night) were documented by clinicians in 96 patients across eight studies and by families in 1012 patients in five studies. Sleep disturbances associated with behavioural outbursts were noted in three clinician‐reported studies and five family‐reported studies. Distress behaviours, including self‐injury and sleep disturbances, were reported in 435 individuals across 10 studies (three clinician‐reported and seven family‐reported). A similar proportion of patients experienced episodic low mood, reported in 467 individuals across three studies.

### Misdiagnosis of epilepsy

Differences between clinician‐reported and family‐reported observations of epileptic and non‐epileptic events were documented by several studies. Reviewing the description of 199 episodes reported as seizures by parents or carers in 60 individuals with RTT, Cardoza et al.^10^ judged only 93 of 199 (47%) to be epileptic in nature. Several studies used video electroencephalography (EEG) or polygraphy to clarify the nature of reported events. In a telemetry study capturing 28 individuals with RTT with ‘typical seizures’, 23 of 28 (82%) episodes were confirmed not to be epileptic.^22^ In this same study, only 5 of 13 electroclinical seizures were recognized as such by parents. A further telemetry study describing two children experiencing seizures considered to be epileptic, found instead that these were episodes of breathing disturbance and motor activity, with one child experiencing generalized tonic–clonic seizures mistaken for episodes of breathing disturbance.^8^ In the Rett Natural History Study (2006–2015), a disagreement between clinicians and carers as to whether episodes were epileptic seizures or not occurred 8% of the time.^23^ In 2.7% of cases, clinicians diagnosed paroxysmal episodes as epileptic seizures that parents believed were not seizures, with the reverse being true in 3.7% of episodes. In an earlier report of the Rare Disease Consortium Research Network for RTT, only 291 of 360 (81%) individuals with RTT whose carers reported a history of epilepsy were felt to have experienced epileptic seizures after an expert evaluation of the clinical description of these events.^24^ Taken collectively, it is possible to both underdiagnose and overdiagnose epileptic seizures in individuals with RTT, with video telemetry consequently proving invaluable.

### Paroxysmal electroencephalography activity

In 8 of 16 females with RTT, Elian and De Rudolf identified a pseudoperiodic EEG pattern, with high‐amplitude slow waves during apnoea and faster rhythms during hyperventilation or normal breathing. This was interpreted as ‘behaviourally determined respiratory dysrhythmia’.^25^ Whitney et al.^26^ described a 9‐year‐old female with RTT exhibiting self‐sustained paroxysmal alpha activity (9–10 Hz, lasting 1–1.5 seconds) during both wakefulness and non‐rapid eye movement sleep, without clinical correlates or epileptiform features, representing a new non‐epileptic EEG signature.^26^ Another study reported five patients with unilateral rhythmic hand tapping associated with contralateral centrotemporal spikes on EEG.^27^ These episodes were abolished by interrupting hand contact and did not respond to antiepileptic therapy, supporting a non‐epileptic origin. A recent study of 32 patients with RTT described widespread EEG abnormalities, including a new sensorimotor frequency rate (SM_FrR) index that correlated with clinical severity and breathing irregularities.^28^ Collectively, these reports highlight a spectrum of non‐epileptic paroxysmal EEG phenomena in RTT that may further complicate the differentiation of epileptic seizures from non‐epileptic events.

### Rett episodes

Cardoza et al.^10^ defined Rett episodes as ‘non‐epileptic vacant spells’, accompanied by a variety of other features, including motor activity (e.g. twitching, jerking, head turning, falling forward, trembling), autonomic changes (e.g. pupil dilatation, breath‐holding, hyperventilation), and behavioural signs (e.g. staring or laughing). In a study from the British Isles Rett Syndrome Survey, 60 of 89 (67%) respondents were considered to have Rett episodes. In a further UK study of 91 individuals with RTT, Cianfaglione et al.^9^ described Rett episodes in 66 of 91 (73%). Rett episodes were defined as ‘non‐epileptic behaviour often misidentified as possible seizure in which the eye gaze is not fixed and the person appears not to be breathing, with absence of hand movement and motor activities’. Similar episodes have been described by other authors without the explicit use of the term Rett episodes. Glaze et al.^29^ described non‐epileptic episodes as including features of motor, behavioural, and autonomic features, including intermittent breath‐holding and hyperventilation, vacant episodes, inappropriate laughing and screaming, and motor abnormalities such as tremor and dystonia. Julu et al.^14^ reported vacant spells in 48 of 56 children and young people with RTT, associated with involuntary movements and dystonic postures occurring during episodes of differing patterns of abnormal breathing. Sansom et al.^30^ described brief ‘attacks’ of hyperventilation associated with altered consciousness which were not epileptic but were a cause for concern to families in 34 of 107 (32%) children and young people with RTT.^30^

### Methodological quality assessment

Total scores across the eight Murad domains for the 62 papers included in this review ranged from 0 to 8 (median = 4). Only two studies (3.2%) reached high quality (score ≥6), 24 (38.7%) were moderate (scores 4–5), and 36 (68%) were low (≤3).

Representative case selection was a weak‐performing domain, with only 7 of 62 studies (11%) scoring ‘Yes’ on this criterion. In nearly all cases, the patient(s) described were not drawn from a systematically defined or unbiased source population. Most reports originated from tertiary care centres or reflected convenience sampling, often selected for their clinical novelty. The lack of representativeness limits the generalizability of reported findings and reinforces the need for population‐based data in RTT research.

A total of 43 of 62 studies (69%) scored ‘Yes’ on item 2 of the Murad checklist, which assesses whether the exposure (i.e. the presence and nature of non‐epileptic events) was adequately ascertained. The remaining studies either lacked detail on their ascertainment methods or relied solely on retrospective or vague descriptions, limiting confidence in the consistency and accuracy of the reported events. Sensitivity analysis stratified according to study quality (Murad framework) demonstrated that the overall spectrum of non‐epileptic paroxysmal events was broadly consistent across tiers (Table S4).

## DISCUSSION

Our systematic review highlighted the range and diversity of non‐epileptic paroxysmal phenomena observed in individuals with RTT. Respiratory abnormalities were common, with prominent neurological and behavioural disturbances, including vacant spells, dystonic posturing, and episodic agitation. The term Rett episodes has been used variably to describe episodes with more complex phenomenology, often including vacancy, changes in breathing pattern, and either increases or decreases in motor activity.

Accurate differentiation between seizures and non‐epileptic spells is crucial to avoid inappropriate treatment and unnecessary lifestyle restriction. We identified studies describing a misdiagnosis of non‐epileptic events as epileptic seizures in up to 80% of episodes. Such misdiagnosis is not unique to individuals with RTT and is described in 25% to 35% of children and young people referred to tertiary epilepsy clinics.^31^, ^32^ A focus on excluding epilepsy when evaluating paroxysmal episodes is entirely understandable when considering that the point prevalence of epilepsy in individuals with RTT is 30% to 42%, with an overall lifetime prevalence as high as 90%.^23^

Respiratory abnormalities were the most frequently reported non‐epileptic paroxysmal events in RTT, which is an unsurprising finding given that awake breathing disturbances constitute a minor criteria for the diagnosis of RTT.^2^ Prevalence estimates for paroxysmal respiratory disturbances ranged from 24% to 70% of individuals with RTT.^17^, ^33^, ^34^ Hyperventilation often presents in clusters of rapid, deep‐breathing episodes that can alternate with breath‐holding or apnoeic pauses. Breath‐holding episodes, reported in at least 35 studies, may be voluntary or involuntary, occur spontaneously or in response to emotional distress, be central or obstructive in nature, and can occur during wakefulness or sleep.^18^, ^35^ Respiratory disturbances are frequently associated with autonomic dysfunction, with fluctuations in heart rate and blood pressure.^14^ Behaviour manifestations (e.g. agitation, vacant spells, or distress) often accompany these episodes, confounding differentiation from epileptic seizures.^36^

Dystonia, involuntary movements, and paroxysmal limb movements were frequently observed non‐epileptic events in RTT. After the near universally observed stereotypies, dystonia is the most common movement disorder observed in individuals with RTT.^37^ While often present as a more constant background phenomenology, sudden paroxysmal worsening of dystonia may occur, with life‐threatening events identified in our review.^19^ These episodes were reported as having been misdiagnosed as epileptic seizures and appeared to subsequently be responsive to trihexyphenidyl. This highlights the importance of precisely phenotyping non‐epileptic events to ensure that potentially efficacious interventions are not overlooked.

Behavioural disturbances are a common feature of RTT, the phenomenology of which may overlap with other non‐epileptic paroxysmal events. Distress behaviours associated with hyperventilation, including frightened expressions, self‐injury, and screaming were reported in up to 75% of individuals with RTT.^36^ Episodes of daytime and night time screaming and laughing, often occurring unpredictably, have also been described.^10^, ^38^, ^39^ The reported frequency of these episodes was unsurprising as they represent another of the current minor criteria for RTT.^2^ These behaviours are frequently accompanied by autonomic dysfunction, such as changes in breathing patterns or heart rate fluctuations, contributing to diagnostic confusion with epilepsy. Additionally, behavioural episodes can be triggered by environmental or emotional stimuli, making them more variable than epileptic seizures, which often follow a more stereotyped pattern.

Episodes considered by caregivers or clinicians as ‘seizures’ often had no EEG correlation, while conversely epileptic seizures were not recognized. We would recommend caution when approaching the diagnosis of paroxysmal events in individuals with RTT. Initially, a careful description should establish the semiology. We recommend a low threshold for the use of video EEG or prolonged telemetry to capture typical episodes, particularly when caregiver and clinician impressions diverge. This may reduce diagnostic uncertainty, prevent unnecessary treatment escalation, and facilitate more appropriate management. Where formal video EEG is not feasible, caregiver smartphone recordings of events can provide valuable supplementary information for clinicians. For respiratory abnormalities, objective physiological monitoring should be used when available. Techniques such as polygraphic recording, pulse oximetry with event annotation, inductance plethysmography for breath pattern analysis, and transcutaneous CO_2_ monitoring may differentiate causes of apnoea, hyperventilation, and breath‐holding. For motor phenomena such as dystonia, involuntary movements, or paroxysmal limb movements, systematic video documentation, and review may avoid the need for EEG.

Individuals with RTT may experience the range of paroxysmal episodes outlined in Figure 1. Episodes with mixed, more complex, phenomenology may also be experienced. Some authors have focused on distinguishing these events from epileptic seizures.^8^, ^22^ While describing these events as non‐epileptic has benefits in terms of discouraging the use of inappropriate antiepileptic medications, there are limitations defining episodes by what they are not, and greater benefits guiding management through defining what they are. The term Rett episodes was used by a small number of studies identified by our review. We identified two conflicting definitions for Rett episodes.^9^, ^10^ While authors of both studies noted that events include altered responsiveness and may be mistaken for epileptic seizures, the definition proposed by Cardoza et al.^10^ includes a variety of other motor, respiratory, and behavioural features while Cianfaglione et al.^9^ restricted these features to the apparent absence of respiratory effort and an absence of motor activity. Comparable episodes described by other authors have included a range of additional phenomena.^22^, ^30^, ^40^ Smeets et al.^16^ reported an individual with RTT with abnormal spontaneous brainstem activations, precipitated by Valsalva‐type breathing and tachypnoea, and associated with vacant spells and pseudoseizures. These episodes lacked epileptiform correlates on EEG and were interpreted as autonomic and brainstem‐driven events, successfully reduced using carbogen therapy. Abnormal spontaneous brainstem activation‐related events may represent the same clinical phenomena described elsewhere as Rett episodes. A currently unresolved question is whether these descriptions are ultimately of a single form of attack or episode with a unifying pathophysiology, or instead of distinct separate phenomena for which a more precise delineation may guide management. This is a challenging question to answer in a situation where clinical phenomenology is yet to be consistently established. We recommend at present avoiding the term Rett episodes in clinical communication and instead describing events phenomenologically (e.g. respiratory pattern change, altered responsiveness, dystonic posturing, etc).

**Figure 2:**
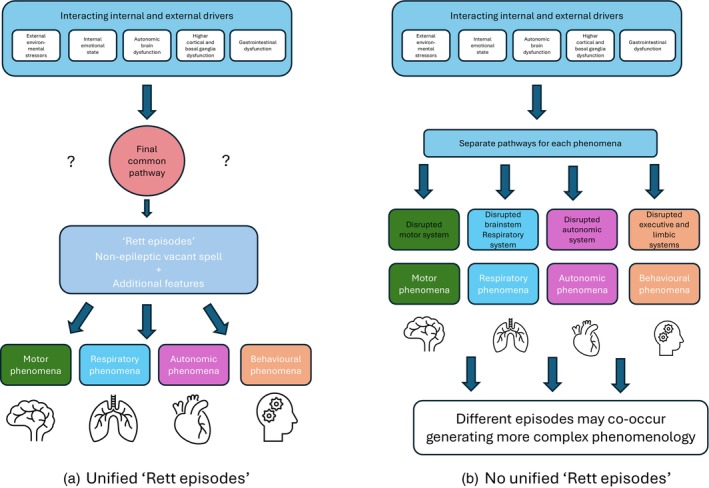
Potential pathways for Rett episodes with an assumption that events arise from a single common pathway (a) and a model of separate pathways independently giving rise to different episodes (b), which may temporally coincide.

RTT is a widespread synaptic disorder, effecting all neurotransmitter systems.^41^ Dysfunction in different neuronal systems and circuitry are likely to result in different aspects of the neurological phenotype (e.g. brainstem autonomic dysfunction accounting for respiratory drive dysfunction, basal ganglia dysfunction resulting in dystonia and stereotypies, and thalamocortical dysfunction explaining the increased incidence of epilepsy). Do Rett episodes arise from dysfunction across these varying neuronal systems, ultimately culminating in dysfunction through an undetermined common pathway resulting in episodes with different phenomenological expression (Figure 2a), or are the pathways subserving different forms of paradoxical episodes entirely sequestered, with different paroxysmal episodes occurring concurrently giving rise to more complex events (Figure 2b)? One construct that may begin to provide a potentially unifying pathophysiology is emotional, behavioural, and autonomic dysregulation.^42^ Considering RTT to be primarily a condition of congenital dysautonomia, Singh and Santosh^42^ proposed that dysregulation of behaviour, emotion, and autonomic dysfunction are three intimately linked and overlapping aspects of a unified disturbance of brain function accounting for much of the pathological issues encountered in RTT. This construct would not fully explain dystonia and other movement disorders experienced by individuals with RTT and also does not explicitly incorporate primary cortical dysfunction as supported by the range of EEG abnormalities seen in RTT and the high prevalence of epilepsy.

## Limitations of the review

A number of limitations must be acknowledged. During the period over which the identified studies were reported, the definition and classification of RTT has evolved and reported series over time may consequently not consist of entirely comparable cohorts. Our search was conducted from 1966 onwards, coinciding with the first description of RTT, but we acknowledge that diagnostic criteria and awareness of RTT have evolved substantially over this time frame, which may limit the comparability of earlier and later reports. Classification of the events reported is based on an interpretation of the descriptions provided by authors, introducing an unavoidable element of subjectivity. Studies were typically reported retrospectively, consisting of convenience samples. A further limitation is the inclusion of diverse study designs, ranging from single‐case reports to clinical trials. This approach inevitably increases heterogeneity; however, given the rarity of RTT and the limited number of systematically collected data sets, we considered it essential to capture the full spectrum of reported non‐epileptic paroxysmal events. Accordingly, any pooled figures are intended only as descriptive summaries and should not be interpreted as precise prevalence estimates.

We did not include grey literature, which may have introduced publication bias, or description of paroxysmal events, which may have been included in national and international RTT registries or captured in smaller observational theses that are not indexed in major databases.

Restricting our review to after 1999 or to genetically confirmed cases would have excluded 17 studies, which is a substantial proportion of the available literature. RTT is a rare disorder; much of the early clinical characterization, including recognition of non‐epileptic paroxysmal events, dates from reports before 1999. Similarly, atypical cases provide valuable insights into the broader clinical spectrum. While these cohorts may not fully align with current diagnostic criteria, excluding them would risk omitting important historical and phenomenological observations that can continue to inform our present understanding. Therefore, we included both reports from before 1999 and reports of atypical RTT, but acknowledge that evolving diagnostic criteria and incomplete genetic ascertainment reduce comparability across studies.

We did not include stereotypic hand movements within the scope of this review because these are a core diagnostic feature of RTT rather than paroxysmal phenomena. While stereotypies may overlap phenomenologically with certain non‐epileptic events and are an important clinical consideration, they have already been well described in the literature to date.

## CONCLUSIONS

Non‐epileptic paroxysmal events are common in individuals with RTT. Respiratory dysfunction, paroxysmal movements, dystonic episodes, vacant spells, autonomic disturbances, and behavioural abnormalities are highly prevalent and may potentially be misclassified as epileptic seizures, leading to inappropriate treatment. Improving clinician awareness and refining diagnostic criteria for non‐epileptic events is essential in preventing inappropriate interventions, reducing medication‐related adverse effects, and ultimately enhancing quality of life for individuals with RTT and their families. The utility of the term Rett episodes remains to be determined. Future research should prioritize developing clear clinical guidelines and standardized assessment tools to facilitate accurate recognition and management of non‐epileptic paroxysmal events in RTT.

## FUNDING INFORMATION

The authors have no funding sources to declare.

## CONFLICT OF INTEREST STATEMENT

DEL has undertaken consultancy work for Acadia and Neurogene.

## Supporting information


**Figure S1:** Literature search and selection flow diagram.

**Table S1:** Database search strategies.

**Table S2:** Data extraction table.

**Table S3:** Study quality and risk of bias: Assessment using the Murad framework.

**Table S4:** Sensitivity analysis of non‐epileptic paroxysmal events in Rett syndrome, stratified by study quality (Murad framework).

## Data Availability

The data that supports the findings of this study are available in the supplementary material of this article.
