# Supplementary figures and images for: Non‐epileptic paroxysmal events in Rett syndrome: A systematic review of case‐based and observational evidence
Source: Dev Med Child Neurol. 2025 Nov 24;68(6):746–54. doi: 10.1111/dmcn.70093 (PMC13160399; doi:10.1111/dmcn.70093)

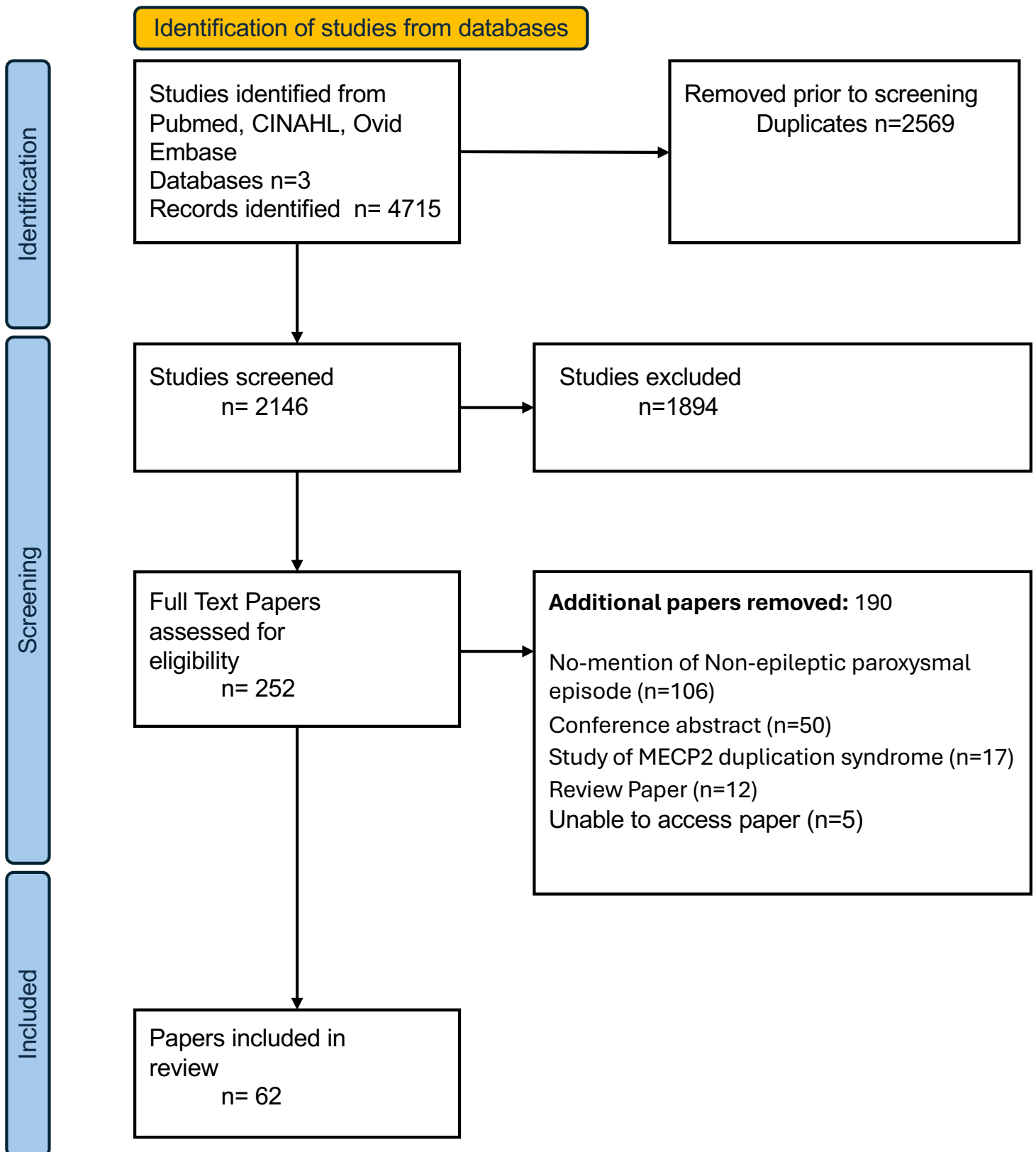

Supplement: Supplementary file 1 — Figure S1: Literature search and selection flow diagram. [file DMCN-68-746-s005.pdf]
