# Supplementary material for: Non‐epileptic paroxysmal events in Rett syndrome: A systematic review of case‐based and observational evidence
Source: Dev Med Child Neurol. 2025 Nov 24;68(6):746–54. doi: 10.1111/dmcn.70093 (PMC13160399; doi:10.1111/dmcn.70093)
Supplement: Supplementary file 2 — Table S1: Database search strategies. [file DMCN-68-746-s003.doc]

**Table S1. Database Search Strategies**

**Search terms and number of results returned from each database (PubMed, Ovid Medline & Embase, and CINAHL) during the systematic literature search on non-epileptic and paroxysmal events in Rett syndrome.**

|  |
| --- |

| Search term | Pubmed | Ovid Medline & Embase | CINAHL |
| --- | --- | --- | --- |
| Rett AND Paroxysmal | 25 | 61 | 2 |
| MECP2 AND Paroxysmal | 6 | 21 | 1 |
| Rett AND non-seizure | 2 | 6 | 0 |
| MECP2 AND non-seizure | 0 | 0 | 0 |
| Rett AND non-epileptic | 9 | 28 | 1 |
| MECP2 AND non-epileptic | 2 | 11 | 0 |
| Rett AND Valsalva | 5 | 17 | 3 |
| Rett AND Epilepsy | 588 | 1617 | 90 |
| MECP2 AND Epilepsy | 278 | 799 | 30 |
| Rett AND Attack | 24 | 24 | 2 |
| MECP2 AND attack | 10 | 15 | 0 |
| Rett AND episode | 74 | 13 | 10 |
| MECP2 AND episode | 32 | 4 | 4 |
| **Rett AND Spell** | 11 | 5 | 2 |
| MECP2 AND Spell | 5 | 4 | 2 |
| Rett AND Autonomic episode | 4 | 0 | 0 |
| MECP2 AND Autonomic episode | 1 | 0 | 0 |
| | **Rett AND Breath-holding** |  |  |  | | --- | --- | --- | --- | | 19 | 75 | 6 |
| | **MECP2 AND Breath-holding** |  |  |  | | --- | --- | --- | --- | | 3 | 28 | 2 |
| | **Rett AND (Apnoea OR Apnea)** |  |  |  | | --- | --- | --- | --- | | 77 | 417 | 17 |
| | **MECP2 AND (Apnoea OR Apnea)** | | --- | | 28 | 191 | 4 |
| Total | 1203 | 3336 | 176 |

Total from table: 4715

Total from Endnote: 3780
