# Supplementary material for: Non‐epileptic paroxysmal events in Rett syndrome: A systematic review of case‐based and observational evidence
Source: Dev Med Child Neurol. 2025 Nov 24;68(6):746–54. doi: 10.1111/dmcn.70093 (PMC13160399; doi:10.1111/dmcn.70093)
Supplement: Supplementary file 3 — Table S2: Data extraction table. [file DMCN-68-746-s004.doc]

**Table S2. Data Extraction Table**

Summary of Study Characteristics and Extracted Non-Epileptic Paroxysmal Events in Individuals with Rett Syndrome

| Author | Number of patients | Mean Age   or Range | Neuroglial | Respiratory | Behavioural | Patient/family vs clinician reported |
| --- | --- | --- | --- | --- | --- | --- |
| Brunel and Gilly (1985) | 1 | Age = 2 years |  | Hyperventilation (n=1) |  | Clinician |
| Lugaresi et al. (1985) | 4 | Mean = 13 years | . | Apnoea (n=4), Hyperventilation (1) |  | Clinician |
| (Cirignotta et al., 1986) | 4 | Range = 8 - 21 years | . | Hyperventilation (n=4) Apnoea (n=4) |  | Clinician |
| Verma et al. (1986) | 9 | Range = 2-15 |  | Hyperventilation (n=7) |  | Clinician |
| (Wu et al., 1988) | 9 | Range: 20 months - 3 years |  | Hyperventilation (n=9) | .Behavioural changes (n=2) | Clinician |
| Southall et al. (1988) | 18 | Range= 6-17 years |  | Hyperventilation (n=10) Apnoea (n=10), Valsalva (n=14) | . | Clinician |
| (Garofalo et al., 1988) | 9 | NS |  | Hyperpnea preceding apnea (n=2) | Episodic behavioural changes (n=2) | Clinician |
| (Kerr et al., 1990) | 14 | Mean=7 years | Paroxysmal limb movement with respiratory dysrhythmia (n=5) EEG paroxysms at 1.5-4 Hz (n=6) | Hyperventilation (n=10) Apnoea (n=14) |  | Clinician |
| Bruck et al 1990 | 7 | Range = 2-10 years |  | Hyperventilation (n=3) |  | Clinician |
| Elian and De Rudolf (1991) | 16 | Range = 8 months – 20 years | Pseudo-periodic EEG patterns linked to alternating apnoea and hyperventilation (n=8) | Apnoea and hyperventilation (n=8) |  | Clinician |
| (Witt Engerström, 1992) | 91 | median age 17 1/2 years |  | **Hyperventilation (n=**22) | **Screaming/laughter spells (n=**24) | NS |
| Matsuishi et al. (1992) | 7 | NS |  | **Breath holding (n=7)** |  | Clinician |
| Sansom et al. (1993) | 107 t | Mean age= 10.6 years | Altered consciousness associated with hyperventilation (n=34), Teeth grinding (n=1) | Hyperventilation (n=34), Apnoea (n=1) | Self injurious behaviour (n=52), Night-time laughing n=90, Crying: (n=63), Screaming (n=52), Episodes of anxiety (n=81) | Clinician |
| Marcus et al. (1994) | 13 | Mean = 8.08 years |  | "Clician reported Hyperventilation (n=20) Apnoea (n=20), Family reported Hyperventilation: n = 10 Apnoea / breathholding spells: n = 9 Cyanosis: n = 6" | . | Clinician & Family |
| Glaze et al. (1998) | 82 | mean = 7–8 years | Non epileptic seizure events (n=23) : n= NS ( Involuntary Eye Movements ,  Vacant spells, Paroxysmal limb movement  Head Movements  Stiffening  Arrest/falling  Motor ) Tremor |  | Behavioural changes NS | Clinician |
| (Cooper et al., 1998) | 78 | Range = 10 months - 23 years | Vacant spells (n=11), Grimacing nNS | Hyperventilation (n=7) Apnoea (n=7) |  | Clinician |
| (Murakami et al., 1998) | 6 | NS |  | Hyperventilation (n=6), Breath holding (n=6) |  | Clinician |
| Triki and Mhiri (1999) | 5 | NS |  | Apnoea and Hyperventilation (n=1) |  | Clinician |
| (Morton et al., 2000) | 7 | Range = 4 - 33 years |  | Breath holding (n=17), Hyperventilation (n=3) |  | Clinician |
| Julu et al. (2001) | 56 | Mean = 13.2 years | Vacant spells = (n=56) associated with dystonic posturing and abnormal movements (n=48) | Valsalva breathing (=26) Hypopnea, apnoea (n=NS) |  | Clinician |
| Kurihara, Kumagai and Nakae (2001) | 1 | 6 |  | Apnoea (n=NS) |  | Clinician |
| Bruck et al (2001) | 28 | Mean 6.7 years | Teeth grinding (n=6) | Respiratory dysfunction (n=15) | Laughing/shouting spells (n=4), Sleep disturbances (n=7) | Family |
| (Mount et al., 2002) |  |  | Repetitive tongue movements (n = 81), Vacant “staring” spells (n = 112) | Hyperventilation (n = 108)  Breath-holding (n = 100), Air/saliva expelled (air-puffing) (n = 78), Swallows air (aerophagia) (n = 82) | Laughter spells (day) (n = 129)  Laughter spells (night) (n = 91)  Inconsolable crying (day) (n = 95)  Inconsolable crying (night) (n = 61)  Screaming spells (day) (n = 89)  Screams hysterically, cannot be consoled (n = 69)  Screaming spells (night) (n = 65)  Panic spells (n = 104) | Family |
| Cass et al. (2003) | 87 | Range = 2 years 1 month to 44 years 10 months |  | Breathing abnormalities (n= 56 ) Air swallowing (n=20  Hyperventilation (n=50 ) Apnoea (n=34) |  | Clinician |
| Julu and Witt Engerström (2005) | 72 | Mean = 17.3 years | Paroxysmal limb movement associated with involuntary Eye Movements (n=NS), Dysautonomia n=NS | Respiratory Dysrhythmia, Hypopnea NS | . | Clinician |
| Smeets et al. (2006) | 1 | NS | Abnormal spontaneous brainstem activation ASBA (n=1) | Forceful/irregular breathing, Valsalva pattern, tachypnoea , hypocapnoea (n=1) |  | Clinician |
| Huppke et al. (2007) | 110 | Median (n=10 years) |  | Hyperventilation (n=2) | . | Clinician |
| (Rohdin et al., 2007) | 12 | NS |  | Apnoea, Hypoventilation, Shallow breathing (n=12) |  | Clinician |
| Oddy et al. (2007) | 201 | Range = 2-29 |  | Breath-holding and hyperventilation (n=NS) |  | Family |
| Nava et al (2008) | 1 | 9 |  |  | Self injurious behaviour (n=1) |  |
| Vignoli et al. (2009) | 30 | Mean = 18.6 | Myoclonus (n=4), Tremor (n=4), Period Limb Movements and Jerking Movements (n=3) , teeth grinding (n=6) | . |  | Clinician |
| (d'Orsi et al., 2009a) | 1 | 8 |  | Hyperventilation (n=1) Apnoea (n=1) | . | Clinician |
| (d'Orsi et al., 2009b) | 1 | 3 |  | Hyperventilation (n=1) Apnoea (n=1) | . | Clinician |
| Gika et al. (2010), | 3 | Mean = 14 years | Paroxysmal dystonia (n=3) | Apnoea and cyanosis (n=2) | . | Clinician |
| Glaze et al. (2010) | 360 | Range = 8 months to 64 years. | Non Epileptic seizures (n=311) | . |  | Clinician evaluation of parent report |
| Cardoza et al. (2011) | 89 | Range = 5-43 | Vacant spells (n=66) | Apnoea, Hyperventilation, Air swallowing (n=73) |  | Clinician evaluation of parent report |
| ( D'Orsi et al., 2012) | 8 | Mean = 14.5 years | Myoclonus (n=1) | Hyperventilation (n=8) Apnoea (n=8) | . | Clinician |
| Bebbington et al. (2012) | 974 | Range = 1 year 4 months - 49 years | Teeth grinding (n=1) | Breathing abnormalities, Air swallowing, Hyperventilation (n=NS) | sleep disturbance (n=1). Alterations in mood, with day and night screaming and laughing (2) | Clinician |
| (Vignoli et al., 2012) | 84 families | Mean = 24 years |  | Apnea (n=48)  Hyperventilation (n= 30), Heart arrhythmias (n=15) - not clear if there’s a clinical correlation | Behavioural disturbance and episodic agitation (n=58) | Clinician |
| (Gokben et al., 2012) | 1 | 11 years old |  | Hyperventilation, Apnea, cyanosis (n=1) | Behavioural disturbance (n=1) | Clinician |
| (Carotenuto et al., 2013) | 13 | Mean age = 8.08 | Paroxysmal limb movement (n=13) | Hypopnea (n=13) |  | Clinician |
| Bao et al. (2013) | 685 | Mean = 11 years 1 month | Vacant spells (n=40 clinician reported, n=319 parent reported) | Apnoea, Cyanosis (n=40) |  | Clinician and family reported |
| Nissenkorn and Ben-Zeev (2013) | 5 | Mean = 6.2 years | Paroxysmal EEG Activity  (n=5) | . | . | Clinician |
| Whitney et al. (2014) | 1 | 9 | Paroxysmal EEG Activity n= 1 | Apnea (n=1)  Hyperventilation (n= 1), | Sleep disturbances (n=1) | Clinician |
| (Cianfaglione et al., 2015) | 91 | NS | Teeth grinding = (n=52) Rett episodes = (n=66) | Apnoea (n=70) Hyperventilation (n=53) | Sleep disturbances (n=56) | Family |
| Gharesouran et al. (2015) | 1 | 7 |  |  | Sleep disturbance (n=1) | Clinician |
| Boban et al. (2016) | 364 | Range = 2.1–57.2 years |  |  | Night laughing (n=184), night screaming (n=171),  Night waking (n=289) | Family |
| Santosh et al. (2016) | 1 | 13 |  | Hyperventilation (n=1) Apnoea (n=1) | . | Clinician |
| Ohno et al. (2016) | 1 | 11 |  | Apnoea (n=1) |  | Clinician |
| (Pini et al., 2016) | 151 | Mean age = 12 years |  | Apnoea (n=141), forceful breathing (n=66), |  | Clinician |
| (MacKay et al., 2017) | 413 | Range: 2-57 |  | Breath-holding (n-284 |  | Family |
| Mancini et al. (2018) | 34 | Mean = 10.4 years |  | Apnoea n=34 | . | Clinician |
| Tarquinio et al. (2018) | 1,185 | Age = 10.2 years |  | Hyperventilation (n=466), Breath holding (n=605), Air swallowing (n=435), Puffing air/saliva (n= 583) |  | Clinician |
| (Chou et al., 2019) | 23 | Mean = 12.87 years ± 6.62 | Dysautonomia (n=NS) | Respiratory dysrhythmia (n=NS) | . | Clinician |
| (Sarber et al., 2019) | 13 | Mean = 10.3 years |  | Hyperpnea followed by central apnoea during wakefulness (n=13) |  | Clinician |
| Kamdar et al. (2020) | 25 | NS | Teeth grinding (n=13) | Hyperventilation (n=6) Apnoea (n=6) | Sleep disturbance (n=9) Laughter spells (n=7) | Clinician |
| Peron et al. (2022) | range = 19–49 | NS |  | Apnoea (n=27/50) associated with hyperventilation (n=12/50, hyperventilation only (n=7) | Screaming/Crying/Laughing Spells (Day or Night) (n=18) | Family |
| Portnova et al. (2022) | 32 | **Mean age:** **8.46 ± 4.15 years** | EEG abnormalities (n=NS) | Breathing irregularities (n=31) |  | Clinician |
| Akiyama et al. (2023) | 1 | 29 | Myoclonus (n=1) |  |  | Clinician |
| (Cherchi et al., 2023) | 52 | Median - 15 |  | Apnoea (n=42), Hyperventilation (n=12) |  | Family |
| Anushka Bhowal, MD1, Felicia Cooper, MD1 Elizabeth Donner, MD1 (2024) | 1 | 13 |  | Apnoea (n=1) | Crying (n=1) | Clinician |
| (Peri et al., 2024) | 11 | Mean = 13 +/- years |  | Day apnoea (n=1) | Night time behavioural disturbance of creaming and crying (n=NS) | Family and clinician |

| Key |  |
| --- | --- |
| t | Data taken from 107 surveys |
| NS | Not specified |
