# Supplementary material for: Non‐epileptic paroxysmal events in Rett syndrome: A systematic review of case‐based and observational evidence
Source: Dev Med Child Neurol. 2025 Nov 24;68(6):746–54. doi: 10.1111/dmcn.70093 (PMC13160399; doi:10.1111/dmcn.70093)
Supplement: Supplementary file 4 — Table S3: Study quality and risk of bias: Assessment using the Murad framework. [file DMCN-68-746-s002.doc]

**Table S3: Study Quality and Risk of Bias - Assessment Using the Murad Framework**

The Murad tool evaluates eight binary domains across four key areas: selection, ascertainment, causality, and reporting. The domains include representative case selection, exposure ascertained, outcome ascertained, alternative causes ruled out, challenge–rechallenge phenomenon, dose–response relationship, follow-up sufficiency, and level of reporting detail. Each study was scored “Yes” or “No” for each domain, with a maximum possible score of 8. Based on the total score, studies were categorised as high quality (6–8), moderate quality (4–5), or low quality (0–3).

| **Author(s)** | **1. Representative Cases (Selection)** | **2. Exposure Ascertained (Ascertainment)** | **3. Outcome Ascertained**  **(Ascertainment)** | **4. Alternative Causes Ruled Out (Causality)** | **5. Challenge/ Rechallenge (Causality)*** | **6. Dose– Response Effect (Causality)*** | **7. Follow-Up Sufficient (Causality)** | **8. Sufficient Detail (Reporting)** | **Total Score (out of 8)** | **Quality Judgment (High/Moderate/Low)** |
| --- | --- | --- | --- | --- | --- | --- | --- | --- | --- | --- |
| Brunel and Gilly (1985) | **No** | **No** | **Yes** | **Yes** | **NA** | **NA** | **Yes** | **Yes** | **4** | **Moderate** |
| Lugaresi et al. (1985) | **No** | **Yes** | **Yes** | **Yes** | **NA** | **NA** | **Yes** | **Yes** |  | **Moderate** |
| (Cirignotta et al., 1986) | **No** | **Yes** | **Yes** | **No** | **NA** | **NA** | **No** | **Yes** | **3** | **Low** |
| Verma et al. (1986) | **No** | **Yes** | **Yes** | **Yes** | **No** | **No** | **Yes** | **Yes** | **4** | **Moderate** |
| (Wu et al., 1988) | **No** | **No** | **No** | **Yes** | **NA** | **NA** | **No** | **Yes** | **2** | **Low** |
| Southall et al. (1988) | **No** | **Yes** | **Yes** | **Yes** | **NA** | **NA** | **Yes** | **Yes** | **5** | **Moderate** |
| (Garofalo et al., 1988) | **No** | **Yes** | **Yes** | **No** | **NA** | **NA** | **No** | **No** | **2** | **Low** |
| (Kerr et al., 1990) | **No** | **Yes** | **Yes** | **Yes** | **NA** | **NA** | **Yes** | **Yes** | **5** | **Moderate** |
| Bruck et al 1990 | **No** | **No** | **Yes** | **Yes** | **NA** | **NA** | **Yes** | **Yes** | **4** | **Moderate** |
| Elian and De Rudolf (1991) | **No** | **Yes** | **Yes** | **No** | **NA** | **NA** | **Yes** | **Yes** | **2** | **Low** |
| (Witt Engerström, 1992) | **No** | **Yes** | **No** | **No** | **NA** | **NA** | **No** | **No** | **1** | **Low** |
| Matsuishi et al. (1992) | **No** | **Yes** | **Yes** | **No** | **NA** | **NA** | **No** | **No** | **2** | **Low** |
| Sansom et al. (1993) | **No** | **No** | **No** | **Yes** | **NA** | **NA** | **No** | **Yes** | **2** | **Low** |
| Marcus et al. (1994) | **No** | **Yes** | **Yes** | **Yes** | **NA** | **NA** | **No** | **Yes** | **4** | **Moderate** |
| Glaze et al. (1998) | **No** | **Yes** | **Yes** | **Yes** | **NA** | **NA** | **No** | **Yes** | **4** | **Moderate** |
| (Cooper et al., 1998) | **Yes** | **Yes** | **Yes** | **No** | **NA** | **NA** | **Yes** | **Yes** | **5** | **Moderate** |
| (Murakami et al., 1998) | **No** | **Yes** | **Yes** | **No** | **NA** | **NA** | **No** | **No** | **2** | **Low** |
| Triki and Mhiri (1999) | **No** | **No** | **No** | **Yes** | **NA** | **NA** | **No** | **Yes** | **2** | **Low** |
| (Morton et al., 2000) | **No** | **Yes** | **Yes** | **No** | **NA** | **NA** | **No** | **No** | **2** | **Low** |
| Julu et al. (2001) | **No** | **Yes** | **Yes** | **Yes** | **NA** | **NA** | **Yes** | **Yes** | **5** | **Moderate** |
| Kurihara, Kumagai and Nakae (2001) | **No** | **Yes** | **Yes** | **No** | **Yes** | **No** | **No** | **No** | **3** | **Low** |
| Bruck et al (2001) | **No** | **No** | **Yes** | **Yes** | **NA** | **NA** | **Yes** | **Yes** | **4** | **Moderate** |
| (Mount et al., 2002) | **No** | **Yes** | **Yes** | **No** | **NA** | **NA** | **No** | **No** | **2** | **Low** |
| Cass et al. (2003) | **No** | **Yes** | **Yes** | **No** | **NA** | **NA** | **No** | **Yes** | **3** | **Low** |
| Julu and Witt Engerström (2005) | **Yes** | **Yes** | **Yes** | **No** | **Yes** | **NA** | **No** | **Yes** | **5** | **Moderate** |
| Smeets et al. (2006) | **No** | **Yes** | **No** | **No** | **NA** | **NA** | **No** | **No** | **1** | **Low** |
| Huppke et al. (2007) | **Yes** | **Yes** | **Yes** | **Yes** | **Yes** | **NA** | **Yes** | **Yes** | **7** | **High** |
| (Rohdin et al., 2007) | **No** | **Yes** | **Yes** | **No** | **NA** | **NA** | **No** | **No** | **2** | **Low** |
| Oddy et al. (2007) | **No** | **No** | **Yes** | **No** | **NA** | **NA** | **No** | **No** | **1** | **Low** |
| Nava et al (2008) | **No** | **No** | **No** | **No** | **NA** | **Na** | **No** | **No** | **0** | **Low** |
| Vignoli et al. (2009) | **No** | **No** | **Yes** | **No** | **NA** | **NA** | **No** | **Yes** | **2** | **Low** |
| (d'Orsi et al., 2009a) | **No** | **Yes** | **Yes** | **Yes** | **Yes** | **Yes** | **No** | **No** | **6** | **Moderate** |
| (d'Orsi et al., 2009b) | **No** | **Yes** | **Yes** | **Yes** | **NA** | **NA** | **No** | **Yes** | **4** | **Moderate** |
| Gika et al. (2010), | **No** | **Yes** | **Yes** | **Yes** | **NA** | **NA** | **Yes** | **Yes** | **5** | **Moderate** |
| Glaze et al. (2010) | **Yes** | **Yes** | **Yes** | **No** | **NA** | **NA** | **Yes** | **Yes** | **5** | **Moderate** |
| Cardoza et al. (2011) | **Yes** | **No** | **No** | **No** | **NA** | **NA** | **No** | **No** | **1** | **Low** |
| ( D'Orsi et al., 2012) | **No** | **Yes** | **Yes** | **No** | **NA** | **NA** | **No** | **Yes** | **3** | **Low** |
| Bebbington et al. (2012) | **Yes** | **No** | **No** | **No** | **NA** | **NA** | **Yes** | **Yes** | **3** | **Low** |
| (Vignoli et al., 2012) | **No** | **No** | **Yes** | **No** | **NA** | **NA** | **No** | **Yes** | **2** | **Low** |
| (Gokben et al., 2012) | **No** | **Yes** | **Yes** | **No** | **NA** | **NA** | **No** | **No** | **2** | **Low** |
| (Carotenuto et al., 2013) | **No** | **Yes** | **Yes** | **Yes** | **NA** | **NA** | **No** | **Yes** | **4** | **Moderate** |
| Bao et al. (2013) | **No** | **Yes** | **No** | **No** | **NA** | **NA** | **No** | **Yes** | **2** | **Low** |
| Nissenkorn and Ben-Zeev (2013) | **No** | **Yes** | **Yes** | **Yes** | **NA** | **NA** | **No** | **Yes** | **4** | **Moderate** |
| Whitney et al. (2014) | **No** | **Yes** | **Yes** | **No** | **NA** | **NA** | **Yes** | **Yes** | **4** | **Moderate** |
| (Cianfaglione et al., 2015) | **No** | **Yes** | **No** | **No** | **NA** | **NA** | **No** | **Yes** | **2** | **Low** |
| Gharesouran et al. (2015) | **N**o | **No** | **Y**es | **Y**es | **N**A | **N**A | **Y**es | **Y**es | **4** | **Moderate** |
| Boban et al. (2016) | **No** | **No** | **Yes** | **No** | **NA** | **NA** | **No** | **Yes** | **2** | **Low** |
| Santosh et al. (2016) | **No** | **Yes** | **Yes** | **No** | **NA** | **NA** | **No** | **Yes** | **3** | **Low** |
| Ohno et al. (2016) | **No** | **Yes** | **Yes** | No | **NA** | NA | **No** | **No** | **2** | **Low** |
| (Pini et al., 2016) | **No** | **Yes** | **Yes** | No | **NA** | NA | **No** | **No** | **2** | **Low** |
| MacKay et al., 2017 | **Yes** | **No** | **Yes** | No | **NA** | NA | **No** | **Yes** | **3** | **Low** |
| Mancini et al. (2018) | **No** | **Yes** | **Yes** | **Yes** | **Yes** | **Yes** | **Yes** | **Yes** | **7** | **High** |
| Tarquinio et al. (2018) | **Yes** | **Yes** | **Yes** | **No** | **NA** | **NA** | **Yes** | **Yes** | **5** | **Moderate** |
| (Chou et al., 2019) | **No** | **No** | **Yes** | **No** | **NA** | **NA** | **No** | **Yes** | **4** | **Moderate** |
| (Sarber et al., 2019) | **No** | **Yes** | **Yes** | **No** | **NA** | **NA** | **No** | **No** | **2** | **Low** |
| Kamdar et al. (2020) | **No** | **No** | **Yes** | **No** | **NA** | **NA** | **No** | **Yes** | **3** | **Low** |
| Peron et al. (2022) | **No** | **Yes** | **Yes** | **No** | **NA** | **NA** | **Yes** | **Yes** | **4** | **Moderate** |
| Portnova et al. (2022) | **No** | **Yes** | **Yes** | **No** | **NA** | **NA** | **No** | **Yes** | **3** | **Low** |
| Akiyama et al. (2023) | **Yes** | **Yes** | **Yes** | **No** | **NA** | **NA** | **Yes** | **Yes** | **5** | **Moderate** |
| (Cherchi et al., 2023) | **No** | **Yes** | **Yes** | **No** | **NA** | **NA** | **No** | **No** | **2** | **Low** |
| Anushka Bhowal, MD1, Felicia Cooper, MD1 Elizabeth Donner, MD1 (2024) | **No** | **No** | **Yes** | **Yes** | **NA** | **NA** | **Yes** | **Yes** | **4** | **Moderate** |
| (Peri et al., 2024) | **No** | **Yes** | **Yes** | **No** | **NA** | **NA** | **No** | **No** | **2** | **Low** |
