# Supplementary material for: Non‐epileptic paroxysmal events in Rett syndrome: A systematic review of case‐based and observational evidence
Source: Dev Med Child Neurol. 2025 Nov 24;68(6):746–54. doi: 10.1111/dmcn.70093 (PMC13160399; doi:10.1111/dmcn.70093)
Supplement: Supplementary file 5 — Table S4: Sensitivity analysis of non‐epileptic paroxysmal events in Rett syndrome, stratified by study quality (Murad framework). [file DMCN-68-746-s001.doc]

**Table S4 Sensitivity analysis of non-epileptic paroxysmal events in Rett syndrome, stratified by study quality (Murad framework)**

The distribution of non-epileptic paroxysmal events reported in included studies is shown according to study quality (low, moderate, high), as assessed by the Murad tool. Respiratory abnormalities were the most consistently reported phenomena: apnoea/cyanosis (n=25 low-quality, n=13 moderate, n=1 high) and hyperventilation (n=32 low, n=14 moderate, n=1 high) were observed across all quality levels, confirming their robustness as key features. Valsalva breathing was only described in 2 moderate-quality studies. Vacant spells/altered consciousness were described in 3 moderate and 4 low-quality studies, but not in high-quality reports. Motor phenomena such as dystonia (n=2 moderate), myoclonus (n=2 low, n=1 moderate), and periodic limb movements (n=1 low, n=5 moderate) were documented across low- and moderate-quality studies; tremor (n=1 low) and drop episodes (n=1 moderate) were rare. Behavioural episodes such as episodic screaming/laughter spells (n=11 low, n=4 moderate) and sleep disturbance (n=4 low, n=3 moderate) were predominantly described in lower-tier studies, often with less precise definitions.

| Body System | | Event Description | Low Quality studies (n) | Moderate quality studies (n) | High quality studies (n) |
| --- | --- | --- | --- | --- | --- |
| Neurological Episodes | Altered Consciousness & Awareness | Vacant spells and altered consciousness with staring | 4 | 3 | - |
|  |  | Paroxysmal Activity (EEG abnormalities not consistent with epileptic seizures | 1 | 3 | - |
|  |  | Non-Epileptic seizures (details of phenomenology not provided) | - | 2 | - |
|  | Generalised Body Movements | Paroxysmal Dystonia and Dystonic Posturing | - | 2 | - |
|  |  | Tremor | 1 | - | - |
|  |  | Myoclonus | 2 | 1 | - |
|  |  | Period Limb Movements and Jerking Movements | 1 | 5 | - |
|  |  | Stiffening (not related to Dystonia) | - | 1 | - |
|  |  | Drop episodes | - | 1 | - |
|  |  | Other Motor abnormalities (unspecified) | - | 1 | - |
|  | Cranio-cervical Movements | Involuntary Eye Movements | - | 2 | - |
|  |  | Head Turning | - | 1 | - |
|  |  | Grimacing | - | 1 | - |
|  |  | Teeth Grinding | 5 | 1 | - |
| Respiratory and Autonomic Episodes | | Respiratory Dysrhythmia (irregular breathing) | 2 | 2 | - |
|  |  | Hypopnea | - | 2 | - |
|  |  | Valsalva Breathing | - | 2 | - |
|  |  | Air Swallowing | 3 | 1 | - |
|  |  | Apnoea and cyanosis | 25 | 13 | 1 |
|  |  | Hyperventilation | 32 | 14 | 1 |
|  |  | Other breathing abnormalities (e.g. mouth breathing) | 3 | 2 | - |
|  |  | Dysautonomia | 2 | 2 | - |
| Behavioural episodes | | Episodic behavioural changes, including Screaming/Crying/Laughter spells (day or night) | 11 | 4 | - |
|  | | Low mood | 2 | - | - |
|  | | Sleep disturbances | 4 | 3 | - |
|  | | Rett episodes/attacks | 1 | - | - |
|  | | Self-injurious behaviour | 2 | - | - |
